# Quality and Safety Initiatives in a Pediatric and Congenital Heart Surgery Program in a Lowand Middle-Income Country: The Impact of International Collaboration

**DOI:** 10.21470/1678-9741-2025-0123

**Published:** 2025-08-22

**Authors:** Leonardo Augusto Miana, Valdano Manuel, Desiree S. Machado, Bistra Zheleva, Andreas Tsakistos, Steven Schwartz, Glen Van Arsdell, Davi Freitas Tenório, Elizandra Arita, Giuliana Tommasiello, Paula Vincenzi Gaiolla, Nana Miura, Thiana Yamaguchi, Luiz F. Caneo, Marcelo B. Jatene, Fabio B. Jatene

**Affiliations:** 1 Instituto do Coração (InCor), Hospital das Clínicas da Faculdade de Medicina da Universidade de São Paulo, São Paulo, São Paulo, Brazil; 2 Cardiovascular and Thoracic Service, Complexo Hospitalar de Doenças Cardio-Pulmonares Cardeal Dom Alexandre do Nascimento, Luanda, Angola; 3 Cardiovascular and Thoracic Service, Clínica Girassol, Luanda, Angola; 4 D’Or Institute for Research and Education, Rede D’Or, São Paulo, São Paulo, Brazil; 5 Children's HeartLink, Minneapolis, Minnesota, United States of America; 6 Intensive Care Unit, Hospital of Sick Kids, Toronto, Canada; 7 Department of Surgery, David Geffen School of Medicine at the University of California Los Angeles, Los Angeles, California, United States of America; 8 Dell Medical School, University of Texas at Austin, Austin, Texas, United States of America

**Keywords:** Risk Adjustment, Developing Countries, Congenital Heart Defects, Diagnosis-Related Groups, Infant, Newborn Infant.

## Abstract

**Introduction:**

This study assessed the impact of a quality and safety (Q&S) improvement program on outcomes in pediatric and congenital heart surgery (PCHS) through an international non-governmental collaboration in a low-and-middle-income country (LMIC).

**Methods:**

Surgical data from two distinct periods, PRE (January 2016 - December 2019) and POST (January 2020 - May 2024) Q&S implementation, were analyzed. Outcomes included 30-day mortality, urgency status, patient age, and procedure complexity using the Risk Adjustment for Congenital Heart Surgery (RACHS) 1 classification.

**Results:**

A total of 4,297 surgeries were performed: 2,429 in the PRE and 1,868 in the POST era. Overall, 30-day mortality decreased significantly from 7.5% to 5.1% (P = 0.002), reaching 3.1% in 2024. Urgent surgeries increased from 28% to 44% (P < 0.0001), while mortality in elective and urgent cases dropped from 3.9% to 1.7% (P = 0.0007) and from 16.5% to 9.6% (P < 0.0001), respectively. A shift toward more neonatal and infant cases was observed, with significant reductions in mortality in both groups (P = 0.01). Case mix complexity also increased (RACHS categories 3-6), yet mortality declined across all RACHS strata.

**Conclusion:**

The introduction of Q&S initiatives led to marked improvements in PCHS outcomes, even amid growing case complexity and acuity. These findings highlight the value of structured protocols and sustained Q&S efforts and underscore the transformative role of international partnerships in strengthening surgical care in LMICs.

## INTRODUCTION

**Figure:**
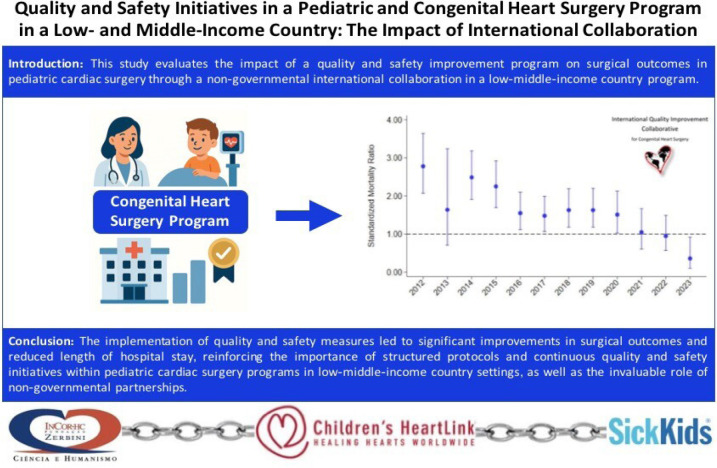

**Table:** 

Abbreviations, Acronyms & Symbols				
CHL	= Children’s HeartLink		M&M	= Morbidity and Mortality
FMUSP	= Faculdade de Medicina da Universidade de São Paulo		NA	= Non-applicable
ICU	= Intensive care unit		NGOs	= Non-governmental organizations
InCor	= Instituto do Coração		PCHS	= Pediatric and congenital heart surgery
IQIC	= International Quality Improvement Collaborative		PRs	= Performance rounds
IQR	= Interquartile range		Q&S	= Quality and safety
LMIC	= Low-and-middle-income country		RACHS	= Risk Adjustment for Congenital Heart Surgery
LOS	= Length of stay		SK	= SickKids

Quality and safety (Q&S) measures are paramount in pediatric and congenital heart surgery (PCHS), a field characterized by high stakes where patient outcomes significantly impact long-term health trajectories^[1,2]^.

Pediatric cardiac programs in low-and-middle-income countries (LMICs) often confront elevated complications and mortality rates, exacerbated by limited investments and Q&S initiatives^[3-5]^. The challenges faced in these regions necessitate innovative solutions to enhance healthcare delivery. One promising approach is developing international collaborations, particularly through non-governmental organizations (NGOs) partnerships, potentially driving meaningful improvements in LMICs healthcare systems^[6-8]^.

Recently, Terao et al.^[9]^ studied success factors and barriers encountered in Quality Improvement Collaboratives in children’s healthcare, finding factors such as data sharing and communication, trust among institutions, financial support, support from national organizations, use of a theoretical framework to guide collaboration, patient and family involvement, and incentives for participation at both the individual and institutional levels to be key drivers to outcomes improvement^[9]^.

High-quality surgical care is essential not only for patient safety but also for ensuring favorable long-term outcomes. The value of Q&S in PCHS cannot be overstated: structured Q&S protocols can mitigate risks associated with surgical procedures, thereby reducing complications and surgically related “never events”^[1,2]^. PCHS in LMICs has additional complex layers, such as limited access to equipment, shortage of specialized medical professionals, and fragile infrastructure^[8]^. Furthermore, socioeconomic factors contribute to limited access to timely intervention or delayed diagnoses^[10,11]^, implicating much-needed healthcare delivery. We sought to describe the impact on the outcomes of a Q&S initiative led by an NGO on our PCHS program.

## METHODS

The study was conducted on the PCHS program of the Instituto do Coração - Faculdade de Medicina da Universidade de São Paulo (InCor-FMUSP) in Brazil. A training and mentoring partnership was established between InCor and The Hospital for Sick Children (SickKids [SK], Toronto, Canada), facilitated by the NGO Children’s HeartLink (CHL).

The collaborative partnership between the North American NGO CHL, SickKids, and the InCor-FMUSP began with an assessment visit in 2017, where the initial evaluation of the InCor’s PCHS program was conducted. This was followed by the formal signing of the partnership agreement in 2018. In the second half of 2018 and the first half of 2019, a multidisciplinary team from InCor visited Toronto SK, comprising one surgeon, one pediatric cardiologist, one anesthesiologist, two intensivists (the intensive care unit [ICU] chief and attending physician), one physiotherapist, and one nurse. Following these visits, findings were presented in team meetings at InCor, and performance rounds (PRs) were implemented to monitor and enhance clinical outcomes. In the second half of 2019, the first official visit from Toronto SK to InCor took place. A team including two intensivists, one pediatric cardiologist/echocardiographer, one surgeon, one nurse, and one physiotherapist stayed for a week, providing on-site feedback and delivering a formal report in November 2019 with recommendations for substantial program improvements. In January 2020, there was an ICU leadership turnover and adoption of new protocols based on partnership recommendations.

During the years 2020 and 2021, due to pandemics restrictions, all live interactions were suspended, but there was an increment in online training. A multidisciplinary team was trained aiming to diminish surgical site infection, and weekly ICU rounds involving members of both teams were implemented.

In the second half of 2022, after the easing of pandemic-related restrictions, a Toronto SK team (consisting of one surgeon, one anesthesiologist, two intensivists, one pediatric cardiologist, and two nurses) revisited InCor. This visit assessed the progress of the Brazilian program and provided new recommendations. In 2023, an InCor team, including one surgeon, one intensivist, two pediatric cardiologists, one imaging specialist (computed tomography/magnetic resonance imaging), one echocardiographer, and one nurse, conducted a one-week visit to Toronto SK. The visit was followed by two comprehensive meetings to discuss findings and maintain momentum in Q&S improvement initiatives. In 2024, due to budget constraints, no in-person visits occurred; however, online case discussions and regular communication channels remained active, sustaining the knowledge exchange and continuous development of the InCor team.

This study was approved by our institutional ethics board (protocol 1.018.019), which waived the requirement for informed consent. Baseline data was collected from 2016 to 2019, referred as the “PRE” era, and compared to outcomes from 2020 to 2024, following the implementation of Q&S initiatives, referred as the “POST” era. All surgical cases performed between 2016 and 2024 at InCor-FMUSP PCHS program were analyzed using the hospital institutional database. Notably, unplanned reoperations performed during the same hospitalization were excluded from this analysis, allowing for a focused examination of index surgeries only.

The primary outcomes analyzed in this study include: 1) the annual number of surgeries performed; 2) surgical complexity stratified by age, urgency status, and Risk Adjustment for Congenital Heart Surgery-1 (RACHS-1)^[12]^ score; 3) 30-day mortality rates and mortality stratified by age, urgency status, and RACHS-1; and 4) postoperative length of stay (LOS).

The protocol implemented in our PCHS program involved significant changes across preoperative, intraoperative, and postoperative procedures. The strategies adopted included:

• Enhanced Preoperative Screening: Surgical case conferences were established to evaluate surgical candidates comprehensively. These conferences provided all PCHS professionals with access to preoperative examinations, fostering collaborative discussions regarding surgical indications and timing. This multidisciplinary approach ensures that diverse perspectives are considered before proceeding to surgical interventions.
• Optimized Case Assignment: Surgical cases were assigned to the most skilled and high-performing surgeons based on data from previous years^[13,14]^, highlighting the correlation between surgeon performance and patient outcomes in congenital heart defects surgeries.
• Surveillance for Residual Lesions: Prompt and continuous monitoring for residual lesions post-surgery was emphasized to reduce the risk of complications, morbidity, and mortality.
• Implementation of Protocols and Care Plans: Clear protocols and care plans were established to guide clinical decision-making, promoting standardized management of children undergoing cardiac surgery.
• Leadership Transition in Postoperative ICU: A complete turnover of the medical team in the postoperative ICU was executed to align with ongoing changes and new protocols. This transition was essential for fostering a culture of accountability and adherence to standardized practices.
• Weekly PRs: Following the SK’s PR model^[15]^, we instituted weekly rounds to evaluate surgical outcomes post-discharge and hospital deaths, both individually and collectively, fostering and environment of continuous learning.
• Morbidity and Mortality (M&M) Meetings: Unexpected outcomes were discussed during monthly M&M meetings, providing a platform for reviewing clinical practices and outcomes.
• Standardized Data Collection: Variables and outcomes obtained from PRs were aggregated to generate feedback metrics evaluating critical process indicators such as cardiopulmonary bypass time, heart ischemia time, technical performance score, ICU LOS, mechanical ventilation duration, incidence of major complications, and vasoactive drug usage through the vasoactive-inotropic score. These metrics are crucial for assessing the effectiveness of surgical interventions and identifying areas of improvement^[13]^.

### Statistical Analysis

Statistical analyses were conducted using IBM SPSS Statistics version 26.0 (IBM Corp., Armonk, NY, USA, 2019) and GraphPad Prism version 9. The normality of continuous variables was assessed using the Shapiro-Wilk test, revealing that they were non-normally distributed. Consequently, continuous data are presented as medians with interquartile ranges (IQR). Ordinal and nominal variables are reported as absolute numbers and percentages. Comparisons between the two eras were made using the Mann-Whitney U test for continuous variables and Fisher's exact test for categorical variables, with a *P* -value < 0.05 considered statistically significant.

## RESULTS

**Fig. 1:**
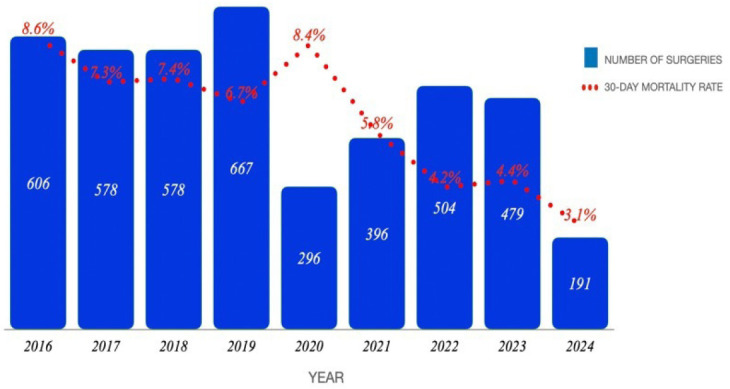
Graph illustrating the number of surgeries and mortality rates per year.

A total of 4,297 surgeries were analyzed, comprising 2,429 conducted in the PRE era (January 2016 - December 2019) and 1,868 procedures performed in the POST era (January 2020 - May 2024). The highest annual mortality rate was recorded in 2016 at 8.4%, while the lowest was observed in 2024 at 3.1% (Figure 1). A continuous trend of decreasing mortality rate was noted, except for 2020, during which a non-significant increase in mortality, compared to previous years, occurred due to a reduction in the volume of elective surgeries (Figure 1). The overall 30-day mortality rate in the PRE era was 7.5%, which significantly decreased to 5.1% following the implementation of Q&S measures ( *P* = 0.002; Table 1).

**Table 1:** Comparison of patients’ demographics, surgical characteristics, and mortality between the PRE and POST eras.

Data	PRE era (n = 2,429)	POST era (n = 1,868)	*P* -value
30-day mortality (%)	182 (7.5%)	96 (5.1%)	0.002
Elective cases (%)	1757/2428 (72.4%)	1055/1868 (56.5%)	< 0.0001
Age	1823	1572	
Neonates	177 (9.7%)	181 (11.5%)	0.09
Infants	551 (30.2%)	652 (41.5%)	< 0.0001
Children	786 (43.1%)	460 (29.3%)	< 0.0001
Adults	309 (17%)	279 (17.7%)	0.55
Neonates + infants	728 (39.9%)	833 (53%)	< 0.001
RACHS-1	1774	1496	
1	386 (22%)	259 (16%)	0.0001
2	551 (31%)	473 (30%)	0.54
3	519 (29%)	483 (31%)	0.36
4	151 (9%)	160 (10%)	0.10
5-6	112 (6%)	121 (8%)	0.11
NA	55 (3%)	76 (5%)	0.01
RACHS-1 3 - 6	782 (45.5%)	764 (51.1%)	0.0016

NA=non-applicable; RACHS=Risk Adjustment for Congenital Heart Surgery

When stratified by urgency status, we observed a notable increase in the incidence of urgent surgeries, from 28% in the PRE to 44% in the POST era ( *P* < 0.0001). Furthermore, there was a substantial reduction in mortality rates following elective surgeries, from 3.9% to 1.7% ( *P* = 0.0007). Similarly, mortality rates decreased from 16.5% to 9.6% for urgent surgeries in the POST era (42% mortality reduction, *P* < 0.0001).

Stratifying by age revealed a shift towards a higher volume of neonatal and infant surgeries in the POST era, accompanied by a decrease in surgical procedures for children aged one to 18 years. Notably, the volume of adult congenital heart disease surgeries remained unchanged (Table 1). Compared to the era before the implementation of the international cooperation program (PRE), there was a significant decrease in neonatal and infant surgical mortality in the POST era ( *P* = 0.01; Table 2). Although there was a trend towards reduced mortality in children and adults, this did not reach statistical significance (Table 2).

**Table 2:** Comparison of mortality rates between the PRE and POST eras by case urgency, age groups, and RACHS-1 categories.

Data	PRE era (n= 2,429)	POST era (n=1,868)	*P* -value
Elective cases mortality (%)	69/1688 (3.9%)	18/1055 (1.7%)	0.0007
Urgent cases mortality (%)	111/671 (16.5%)	78/813 (9.6%)	< 0.0001
Age mortality	1823	1572	
Neonates	46/177 (26%)	27/181 (14.9%)	0.01
Infants	50/551 (9.1%)	32/652 (4.9%)	0.005
Children	24/786 (3.1%)	8/460 (1.7%)	0.19
Adults	10/309 (3.2%)	4/279 (1.4%)	0.18
Neonates + infants	96/728 (15%)	59/833 (7%)	0.001
RACHS-1 mortality	1774	1496	
1	1/386 (0.3%)	0/259 (0%)	0.99
2	22/551 (4%)	5/473 (1.1%)	0.003
3	43/519 (8.3%)	31/483 (6.4%)	0.27
4	22/151 (14.6%)	11/160 (6.9%)	0.04
5-6	24/112 (21.4%)	17/121 (14%)	0.17
NA	10/55 (18.2%)	7/76 (9.2%)	0.18
RACHS-1 1 - 2	23/937 (2.5%)	5/732 (0.7%)	0.006
RACHS-1 3 - 6	89/782 (11.4%)	59/764 (7.7%)	0.015

NA=non-applicable, RACHS=Risk Adjustment for Congenital Heart Surgery

Utilizing the RACHS-1 classification system to evaluate the impact of the Q&S program on varying complexities revealed an inversion in the case mix. There was an increase in more complex cases (RACHS-1 categories 3 to 6) compared to less complex cases (RACHS-1 categories 1 and 2) during the POST era ( *P* = 0.001; Table 1), along with a corresponding reduction in mortality across both groups. Specifically, mortality for RACHS-1 categories 1 and 2 reduced from 2.5% to 0.7% ( *P* = 0.006; Table 2), while mortality for RACHS-1 categories 3 to 6 declined from 11.4% to 7.7% ( *P* = 0.01; Table 2).

**Fig. 2:**
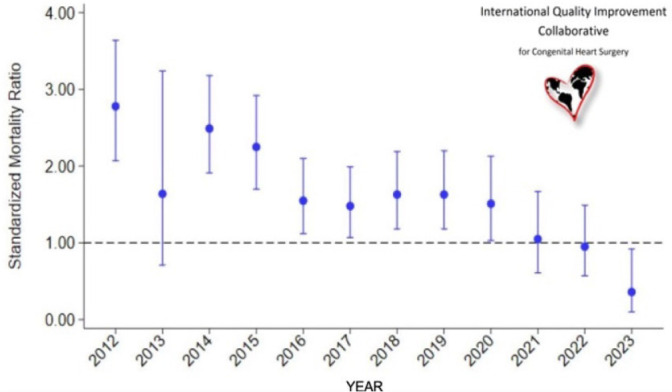
Graph showing standardized mortality ratio comparing our program at Instituto do Coração, Universidade de São Paulo, with the average of International Quality Improvement Collaborative for Congenital Heart Surgery (IQIC) participants from 2012 to 2023. Adapted from IQIC 2024 report.

The Q&S initiative also positively impacted the in-hospital LOS, with a reduction from averages of 12.4 days in the PRE era (IQR = 7.3 - 24.4) compared to 10.1 days in the POST era (IQR = 5.9 - 21.3; *P* < 0.0001; Figure 2). These results collectively demonstrate that the implementation of Q&S initiative led to significant improvements in surgical outcomes, reinforcing the importance of structured measures and continuous quality improvement initiatives alongside the implementation of a culture of safety.

## DISCUSSION

The primary findings of this study indicate a significant reduction in mortality rates following the implementation of a Q&S improvement program developed through international collaboration. Despite initial setbacks due to the coronavirus disease 2019 pandemic, consistent positive outcomes were achieved starting in 2021, with sustained improvements thereafter. The observed reduction in surgical volume appeared to be multifactorial, primarily influenced by challenges in resuming elective surgeries post-pandemic.

Our previous reports highlighted that, despite nearly halting elective surgeries during the pandemic and experiencing increased case complexity, there was no significant rise in mortality compared to the previous year^[16]^. Importantly, starting in 2020, we noted a shift in case mix characterized by an increase in urgent and complex coupled with a reduction in elective surgeries. This shift was partly due to smaller programs absorbing fewer complex cases, which impacted our ability to return to pre-pandemic surgical volumes. Fortunately, the Q&S measures implemented allowed us to mitigate these challenges, decreasing mortality rates for urgent cases and among neonatal and infant patients across various RACHS-1 complexities.

Our program has been part of the International Quality Improvement Collaborative (IQIC), which includes a database to allow LMIC programs to benchmark against each other, and Quality Improvement, a key drivers methodology covering many aspects of patient care^[17]^. The improvement in mortality rates after 2021 is clearly demonstrated by the 2024 IQIC data report, which shows that InCor-FMUSP consistently performed above average after 2021 and maintained its status among the high performers in recent years (Figure 2).

The current Q&S program's emphasis on comprehensive patient preparation - including enhanced preoperative evaluations^[18]^, targeted surgical assignments^[14]^, mandatory intraoperative echocardiography, and elevated safety expectations^[15]^ - has likely contributed to these positive changes. Previous literature underscores the benefits of international collaboration with NGOs, which facilitates knowledge exchange and resource allocation and ultimately leads to improved patient outcomes in pediatric and cardiovascular surgery^[6-8,17]^.

However, it is essential to recognize that improvements may vary across different centers and cultural contexts. The true potential of international collaborations lies in empowering local healthcare teams and fostering a culture of continuous improvement. While substantial transformations can be challenging and time-consuming, our study illustrates that significant progress can be achieved relatively quickly with dedicated efforts^[8,9,17]^.

This study analyzes the impact of Q&S initiatives driven by international collaboration on a high-volume PCHS program within an LMIC setting. The reduction in mortality across all age groups and surgical risk strata indicates the establishment of a safer patient environment. Although pinpointing the most critical factors contributing to these outcomes is complex due to the multifaceted nature of improvements, it is clear that a combination of strategies has played a significant role in enhancing patient care. And although studies conducted in other LMICs have consistently reported the positive impact of international cooperation strategies on healthcare outcome^[6-9]^, none have documented such significant improvements in a short timeframe as observed in our study. We attribute this success to the combination of an established high-volume program and the precise assessment and expertise provided by our partners. By identifying key issues, proposing effective solutions, and ensuring that all initiatives were not only endorsed by local leadership, but also well received at all layers of work.

It is crucial to emphasize that international collaborations rely heavily on voluntary contributions of numerous individuals, as well as institutional interest from LMICs. The humility and commitment of these programs are vital for their success. While economic investment may not be substantial, especially considering the limited financial resources available to these hospitals, investments in organization, culture, and training can lead to transformative changes, as evidenced by this and previous studies^[6-8,19]^. It requires perfect synchronization among stakeholders: well-structured NGOs capable of identifying program needs and recruiting skilled and dedicated volunteers who embrace their roles, and professionals in LMIC programs who humbly acknowledge their own needs and limitations while welcoming new learning and knowledge for the benefit of their patients.

As results materialize, additional stakeholders are likely to join the process of change and improvement, further strengthening these mechanisms. Professional growth within these programs leads to better training and education for new professionals entering the field, creating a multiplier effect that enhances overall capacity and performance^[9,19]^.

### Limitations

This study is a retrospective observational analysis with inherent limitations associated with this design. While comparing an earlier era with a more recent one may partially explain our findings, the robustness of the observed outcomes suggests that the interventions had a substantial impact.

## CONCLUSION

The implementation of Q&S measures led to significant improvements in surgical outcomes and reduced length of hospital stay, reinforcing the importance of structured protocols and continuous Q&S initiatives within pediatric cardiac surgery programs in LMIC settings, as well as the invaluable role of non-governmental partnerships.
